# Septic Bursitis in an 8-Year-Old Boy

**DOI:** 10.1155/2014/823921

**Published:** 2014-05-13

**Authors:** Panagiotis Kratimenos, Ioannis Koutroulis, Dante Marconi, Jennifer Ding, Christos Plakas, Margaret Fisher

**Affiliations:** ^1^Neonatal-Perinatal Medicine, St. Christopher's Hospital for Children, Drexel University College of Medicine, Philadelphia, PA, USA; ^2^Department of Pediatrics, The Unterberg Children's Hospital at Monmouth Medical Center, Drexel University College of Medicine, Long Branch, NJ, USA; ^3^Department of Emergency Medicine, St. Christopher's Hospital for Children, Drexel University College of Medicine, Philadelphia, PA, USA

## Abstract

*Background.* The prepatellar bursa can become inflamed owing to repeated trauma. Prepatellar bursitis is extremely rare in children. *Methods.* We report the case of an 8-year-old boy who was treated for an erythematous, swollen, and severely painful right knee, fever, inability to bear weight on the leg, and purulent material draining from a puncture wound. We describe the differential diagnosis for tender swollen knee, including infection, gout, rheumatoid arthritis, and osteoarthritis. If untreated, prepatellar bursitis can progress to patellar osteomyelitis. *Results.* Wound cultures grew *Streptococcus pyogenes*, with the infection resolving with amoxicillin. *Conclusions.* A high index of suspicion is necessary in children presenting with prepatellar bursitis to prevent potentially devastating sequelae of infection of the septic joint.

## 1. Introduction

A bursa is a synovium-like cellular membrane overlying bony prominences such as subdeltoid, olecranon, ischial, trochanteric, semimembranosus-gastrocnemius, and prepatellar. The prepatellar bursa is located between the patella and the overlying skin and commonly becomes inflamed due to repeated trauma, such as kneeling on hard surfaces, causing bursitis. Prepatellar bursitis often occurs in adults who work in an occupation that requires frequent kneeling, for example, cleaning floors. In fact, prepatellar bursitis has nicknames such as “housemaid's knee” due to this common etiology. Patients with prepatellar bursitis normally have preserved range of motion [1, 2].

## 2. Case Report

An 8-year-old boy, with no significant medical history, presented to the emergency department reporting an erythematous, swollen, and severely painful right knee. Two days prior to his presentation to the emergency department, he was in his usual state of health when he fell off his scooter and scraped his knee on the pavement. He sustained a puncture wound but did not recall any foreign object lodging in his knee. Over the next 2 days, his knee became progressively red, swollen, and tender. He developed a tactile temperature that was not recorded. On the day of admission, he was unable to bear weight on the leg, and purulent material was draining from the wound. As per his mother, this was the child's first trauma and hospitalization.

On admission his vital signs were as follows: temperature 102.3°F, heart rate 102 beats/minute, respiratory rate 38 breaths/minute, blood pressure 102/58 mm Hg, and oxygen saturation 100% in room air. His weight was 52 kg (100th percentile), height was 132 cm (100th percentile), and body mass index was 17.4 (79th percentile).

Physical examination findings were significant for a midline 8 × 10 mm puncture wound on the anterior aspect of the right knee. Purulent material was actively draining from the wound. The knee was erythematous, swollen, and severely tender to palpation. The erythema extended up to the mid femur. There was marked swelling of the lateral and anterior thigh. Joint effusion was not appreciated. The right lower leg and ankle were also edematous. There was mild active and passive movement approximately 10° to 25° in total. Other physical examination findings were unremarkable.

Complete blood count values were as follows: white blood cell count 27.6 K/mm^3^, red blood cell count 4.62 M/mm^3^, hemoglobin 12.3 g/dL, hematocrit 37.3%, platelets 325 K/mm^3^, neutrophils 63%, lymphocytes 14, bands 18, erythrocyte sedimentation rate 68 mm/h, and C-reactive protein 228 mg/L.

Because infectious arthritis was high in the differential diagnosis, wound and blood cultures were obtained and antimicrobial treatment was initiated with intravenous vancomycin and clindamycin and the patient was admitted to the hospital. An orthopedic surgery consult was obtained and explorative arthroscopy was undertaken.

In the operating department, the puncture wound was extended about a centimeter in both directions. Pus was expressed with manual manipulation. There was a moderate amount of pus still under the skin. Sterile cotton swabs were used to break up any adhesions under the skin and to make sure there were no pockets of pus or anything walled off such as an abscess or phlegmon; cultures were sent and the joint was irrigated with approximately 3 L of saline. The capsule was inspected and there was no communication into the joint but some necrotic tissue was excised. A Penrose drain was placed in the bursa and the skin was closed loosely around it.

The patient improved rapidly after surgery. Active and passive movement increased to a total of 90°. Wound cultures grew* Streptococcus pyogenes *sensitive to ampicillin. The vancomycin and clindamycin were discontinued and intravenous ampicillin was started.

The tenderness gradually resolved, range of motion in the joint improved, and the fever resolved. On day 4 of admission, the child was discharged home on amoxicillin with instructions to follow up with pediatric orthopedics in 2 weeks.

## 3. Discussion 

The differential diagnosis for a tender swollen knee includes infection and arthritic conditions such as gout, rheumatoid arthritis, and osteoarthritis. Therefore, thorough workup must be performed to make this diagnosis. Bursitis can be differentiated from septic arthritis and osteomyelitis with a history of more focal tenderness and/or swelling (Table 1). To rule out an infection, joint aspiration is necessary. A synovial fluid white blood cell count greater than 1000/*μ*L suggests infection, rheumatoid arthritis, or gout. Septic arthritis is defined as a white blood cell count greater than 50,000/*μ*L. Once the diagnosis is established, initial management of prepatellar bursitis consists of rest and avoidance of the aggravating factors. Nonsteroidal anti-inflammatory drugs are used to alleviate the inflammation of the bursa. In patients who cannot tolerate nonsteroidal agents, local glucocorticoid injections may be appropriate.

A common consequence of untreated prepatellar bursitis is patellar osteomyelitis. Osteomyelitis is considered a disease of childhood [3]. Because it presents in various ways, diagnosis is often delayed [3]. Osteomyelitis frequently has an unclear course, typically beginning as largely cartilaginous prior to ossification [3]. It is important to prevent bursitis from progressing to osteomyelitis, which can lead to further bony destruction [3]. As a result, a high index of suspicion is necessary in children presenting with prepatellar bursitis initially. Diagnostic tests such as high-quality radiography should be used [3]. Haine et al. [4] explained the importance of magnetic resonance imaging in aiding in the diagnosis as well.

Freys [5] reported a prevalence of septic bursitis as high as three of 1000 patients. In their report the two most frequently infected bursae were the olecranon and the prepatellar. Patients with prepatellar septic bursitis were more likely to be hospitalized than patients with olecranon septic bursitis. The most common organism seen in septic bursitis is* Staphylococcus aureus*, thought to occur in about two-thirds of cases. Other causal organisms include streptococcal species (most commonly group A *β*-hemolytic* Streptococcus*), Gram-negative organisms, and* Mycobacterium marinum*. Raddatz et al. [6] reported that cellulitis adjacent to bursitis occurred in 89% of cases and was often extensive. Also, they found profound edema in 11% of affected extremities. Ten case reports of septic bursitis in children studied over a 25-year period showed a balance between male and female. Eighty percent involved the prepatellar bursa, 80% occurred during the summer months, and 70% required incision and drainage [7]. Temperature, humidity, and local factors as well as bacterial components may favor skin penetration and invasion of superficial bursitis [7]. If the aspiration shows only serous content, then conservative treatment is appropriate with compression, immobilization, antiphlogistics medications (or agents that reduce inflammation, for example, nonsteroidal anti-inflammatory medications), and/or corticosteroids [5]. Typically, patients with a purulent aspiration respond to antibiotics to the targeted organism(s) and to aspirations of the effusions. However, incision and drainage may be necessary if the bursitis does not respond to at least one aspiration [8].

Prepatellar bursitis is extremely rare in children. Searching in PubMed using the words “prepatellar bursitis in children” revealed only two case reports published in 1982. In children, the limited intra-articular joint space and the devastating sequelae of infection of the septic joint decrease our threshold for performing arthrocentesis and sometimes explorative arthroscopy [9, 10].

## Figures and Tables

**Table 1:** Comparison of bursitis, septic arthritis, and osteomyelitis [11–19].

	Clinical	Labs	Microbiology	Imaging
Bursitis	(i) Localized tenderness over area of infection (ii) Decreased range of motion of affected joint or pain with movement (iii) Erythema or edema (iv) History of repetitive movement of involved joint	**Noninfectious** Joint fluid analysis: <2000/*μ*L—predominantly mononuclear cells **Septic** (i) Leukocyte count: mildly to moderately elevated (ii) ESR*: mildly to moderately elevated (iii) Joint fluid analysis: predominantly polymorphonuclear cells (iv) WBC count: 5,000–20,000 *μ*L (possibly >70,000 *μ*L) usually less than septic arthritis (v) Increased protein (vi) Decreased glucose	(i) *S. aureus *most common (>80%) (ii) *Streptococcus* (5–20%)	(i) Plain radiograph and bone scans are not sensitive for bursitis (ii) MRI, if needed, is very sensitive for bursitis

Septic Arthritis	(i) Red, warm, and immobile joint (ii) Often has palpable effusion (iii) Chills and fever occur secondary to bacteremia	(i) Joint fluid; yellow-green color (ii) WBC count >50,000 *μ*L (>75% polymorphonuclear cells) (iii) ESR: elevated	(i) *Staphylococcus* (40%) (ii) *Streptococcus* (30%) (iii) Gram-negative rods (20%)	(i) Plain radiograph: periarticular soft-tissue swelling is most common finding, linear deposition of calcium pyrophosphate (ii) US: used to dx effusions in chronically distorted joints

Osteomyelitis	(i) Swelling, warmth, and erythema over area of infection or affected bone (ii) Painful range of motion of affected joint (iii) Pain in area of infection (iv) Fevers or chills	(i) WBC count: usually does not exceed 15,000 *μ*L and can be normal in chronic osteomyelitis (ii) ESR and CRP** usually increased	(i) Blood cultures positive in only 50% (ii) Most common cause; *Staphylococcus aureus*	(i) Plain radiograph: periosteal thickening or elevation; cortical thickening, sclerosis or irregularity; osteolysis; new bone formation (ii) CT: useful for guiding needle biopsy in closed infections (iii) MRI: gold standard; shows localized marrow abnormalities (iv) US: fluid next to bone without soft-tissue in between usually suggests osteomyelitis (v) Nuclear medicine: 3-phase bone scan helpful for acute stages and shows increased metabolic activity

*ESR (normal values): males (0–15 mm/hr), females (0–20 mm/hr); **CRP (normal value): 0–10 mg/dL.
